# Biogeochemistry of “pristine” freshwater stream and lake systems in the western Canadian Arctic

**DOI:** 10.1007/s10533-016-0252-2

**Published:** 2016-10-11

**Authors:** Joshua F. Dean, Michael F. Billett, Robert Baxter, Kerry J. Dinsmore, Jason S. Lessels, Lorna E. Street, Jens-Arne Subke, Doerthe Tetzlaff, Ian Washbourne, Philip A. Wookey

**Affiliations:** 1 Biological and Environment Sciences, Faculty of Natural Sciences, University of Stirling, Stirling, FK9 4LA UK; 2 Earth and Climate Cluster, Faculty of Earth and Life Sciences, Vrije Universiteit Amsterdam, De Boelelaan 1085, 1081 HV Amsterdam, The Netherlands; 3 School of Biological and Biomedical Sciences, University of Durham, Durham, DH1 3LE UK; 4 Centre for Ecology and Hydrology, Bush Estate, Penicuik, EH26 0QB UK; 5 Northern Rivers Institute, School of Geosciences, University of Aberdeen, Aberdeen, AB24 3UF UK; 6 Environmental Sciences, School of Life Sciences, Heriot-Watt University, Edinburgh, EH14 4AS UK; 7 School of Geosciences, University of Edinburgh, Crew Building, Alexander Crum Brown Road, Edinburgh, EH9 3FF, UK

**Keywords:** Arctic catchments, Inland waters, Freshwater biogeochemistry, Pristine environment, Baseline study, Permafrost thaw, Climate change

## Abstract

Climate change poses a substantial threat to the stability of the Arctic terrestrial carbon (C) pool as warmer air temperatures thaw permafrost and deepen the seasonally-thawed active layer of soils and sediments. Enhanced water flow through this layer may accelerate the transport of C and major cations and anions to streams and lakes. These act as important conduits and reactors for dissolved C within the terrestrial C cycle. It is important for studies to consider these processes in small headwater catchments, which have been identified as hotspots of rapid mineralisation of C sourced from ancient permafrost thaw. In order to better understand the role of inland waters in terrestrial C cycling we characterised the biogeochemistry of the freshwater systems in a *c.* 14 km^2^ study area in the western Canadian Arctic. Sampling took place during the snow-free seasons of 2013 and 2014 for major inorganic solutes, dissolved organic and inorganic C (DOC and DIC, respectively), carbon dioxide (CO_2_) and methane (CH_4_) concentrations from three water type groups: lakes, polygonal pools and streams. These groups displayed differing biogeochemical signatures, indicative of contrasting biogeochemical controls. However, none of the groups showed strong signals of enhanced permafrost thaw during the study seasons. The mean annual air temperature in the region has increased by more than 2.5 °C since 1970, and continued warming will likely affect the aquatic biogeochemistry. This study provides important baseline data for comparison with future studies in a warming Arctic.

## Introduction

Climate change predictions for the Arctic regions cover a number of scenarios: warming only, wetting only and warming and wetting (Zhang et al. 2013). Each of these scenarios indicates intensification of Arctic hydrological regimes, creating more extreme droughts and floods (Déry et al. 2009; Rawlins et al. 2010); this is expected to have a profound influence on subsurface C pools and rates of permafrost loss (Hinzman et al. 2013). Modelled warming scenarios predict that there will be a large (53–66 %) reduction in the areal extent of the upper 2–3 m of permafrost by 2100 (Schuur et al. 2013), albeit with a large range of uncertainty (Jiang et al. 2016), which is expected to result in considerable loss of C from permafrost soils (Schuur et al. 2015; Jiang et al. 2016). Warming conditions in the Arctic are also expected to alter the areal extent of thaw ponds and lakes as seasonal permafrost subsidence increases (Karlsson et al. 2014; Smith et al. 2005; van Huissteden et al. 2011; Liljedahl et al. 2016); these water bodies are associated with high CO_2_ and CH_4_ release to the atmosphere (Negandhi et al. 2013; Tan and Zhuang 2015). Intensification of the hydrological cycle, to which the Arctic is predicted to be more sensitive than other areas of the globe (Bintanja and Selten 2014), is thought to have begun to manifest itself in measurable changes in the C cycle in some Arctic catchments (Vonk et al. 2013), although more studies focusing on both large river systems and small headwater catchments are needed to predict and model long-term change (Holmes et al. 2012; Wrona et al. 2016).

Hydrological and C cycles in the Arctic are closely linked, with surface water accessing contemporary C pools, and groundwater potentially accessing older, deeper C pools as permafrost thaws (Neff et al. 2006; Raymond et al. 2007). There is a general assumption that warming in Arctic regions will increase dissolved organic C (DOC) export to the Arctic Ocean as terrestrial biomass production and hydrological connectivity (both vertical and lateral) increases (e.g. Amon et al. 2012; Tank et al. 2016). In sub-Arctic Sweden for example, permafrost peatland plateaus are associated with low annual DOC export (2–3 g C m^−2^ year^−1^) dominated by the snow melt period (*c.* 70 %), and non-permafrost fens are characterized by much higher DOC export (7 g C m^−2^ year^−1^) due to more sustained annual flow through deeper soil layers that then discharges to streams (Olefeldt and Roulet 2014). However, Striegl et al. (2005) showed that growing season export of DOC decreased significantly between 1978–1980 and 2001–2003 in the Yukon River, Alaska. This was likely caused by the combined effect of increased active layer depth, longer residence times in the subsurface, and microbial mineralisation of DOC in the unfrozen soil and groundwater zone. Hence a large fraction of soil-derived DOC would be either retained or mineralised within the subsurface horizons, reducing DOC transport to the drainage network. This is supported by a controlled leaching experiment that found (regardless of temperature and leaching time) that only small amounts of DOC could be released from tundra soils, and that mobilisation of C occurred largely in the particulate phase (Guo et al. 2007). Hydrological processes controlled by active layer depth in permafrost zones therefore play a key role in mobilising and exporting terrestrial C, but there is limited understanding of these processes at local scales, particularly in headwater catchments (Drake et al. 2015; Holmes et al. 2012). Furthermore, few freshwater biogeochemistry studies in the Arctic include all dissolved C species, generally focusing on DOC, DIC, CO_2_ or CH_4_ individually. Permafrost thaw has, however, been linked to increased aquatic C concentrations and fluxes in all forms: DOC (Olefeldt and Roulet 2014), DIC (Dornblaser and Striegl 2015), CO_2_ (Shirokova et al. 2013), and CH_4_ (Walter et al. 2006; Shirokova et al. 2013). These examples from studies in Northern America and Northern Europe therefore show that there is not currently a consensus on whether progressive deepening of the active layer will lead to an increase or a decrease in riverine DOC concentrations and fluxes.

The major ion biogeochemistry of aquatic systems can provide insight into the source(s), extent and rate of the mobilisation of thawed soil material, and it has been hypothesized that concentrations of weathering-derived ions will increase as thaw depth and thermokarst slumping increases, exposing previously frozen mineral soils (Frey and McClelland 2009; Vonk et al. 2015a). There is already some evidence for this from Alaska (Keller et al. 2010) and Siberia (Frey et al. 2007; Tank et al. 2012).

We carried out a study of specific surface water types in a small catchment area of the western Canadian Arctic to identify spatial patterns and distinctive biogeochemical signatures in all forms of aquatic C and major ions. Previous work in the Siksik Creek catchment found that mineral earth hummocks slow runoff rates to the stream channel by increasing surface roughness, but this is overridden by the higher hydraulic conductivity of the organic-rich inter hummocks during high water-table periods; these processes drive site hydrology in the months following the freshet (Quinton and Marsh 1998, 1999; Quinton et al. 2000). Quinton and Pomeroy (2006) also found that snowmelt dominated surface water chemistry early in the thaw season, but that inputs from soil and decomposing vegetation in the conductive inter-hummocks became more important as the season progressed, coupled with inputs from the more calcareous streambed material during low flows. It is not known whether the hydrological influence of the hummock/inter-hummock geomorphology extends to the lake systems in the region. Furthermore, the vegetation in this area has altered substantially in the last three decades through “shrubification” (increases in tall shrub tundra and alder density of 68 and 35 % respectively; Lantz et al. 2013), and there are few (if any) studies linking shrubification to C export in the hydrological system.

In this study we hypothesise that hydrologically distinct water types will differ geochemically on the basis of biogeochemical processes and hydrological connections—which control aquatic C (CO_2_, CH_4_, DOC and DIC) and major ion concentrations—that are likely to be sensitive to future climate change. We aim to test this hypothesis by (1) describing the geochemistry of the water types sampled in the study area and (2) linking the differences observed to specific biogeochemical processes in the terrestrial-aquatic continuum, particularly the presence or absence of enhanced seasonal permafrost thaw. Apart from some earlier research in Siksik Creek (Quinton and Marsh 1998, 1999; Quinton et al. 2000; Quinton and Pomeroy 2006) this is the first time that the biogeochemistry of the wider aquatic system has been characterised in this part of the western Canadian Arctic. Hence this study forms an important platform for future research; this might include the potential impacts of the new Inuvik-Tuktoyaktuk road, which runs within 500 m of the study area and construction of which is currently taking place in part of the study watershed.

## Study site

### Location and climate

**Fig. 1:**
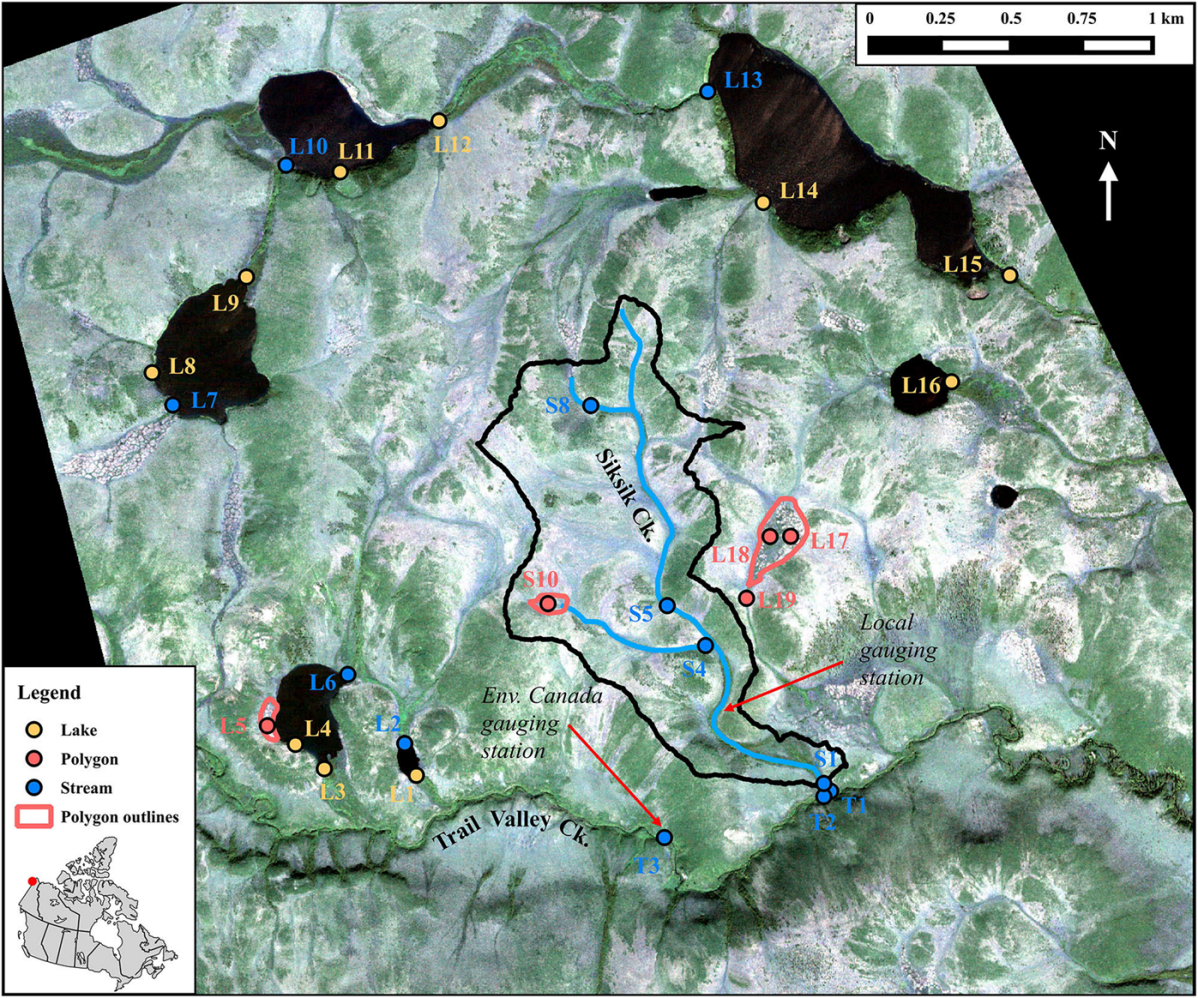
The study region (68°44′54.5″N, 133°29′41.7″W) and sampling sites for the water types identified in this study. Siksik catchment, flow gauging stations and areas of sub-sampled polygonal ice wedges are also shown (for clarity, not all areas of polygonal tundra are identified here). *Inset* location of the study site in the Northwest Territories, Canada

**Fig. 2:**
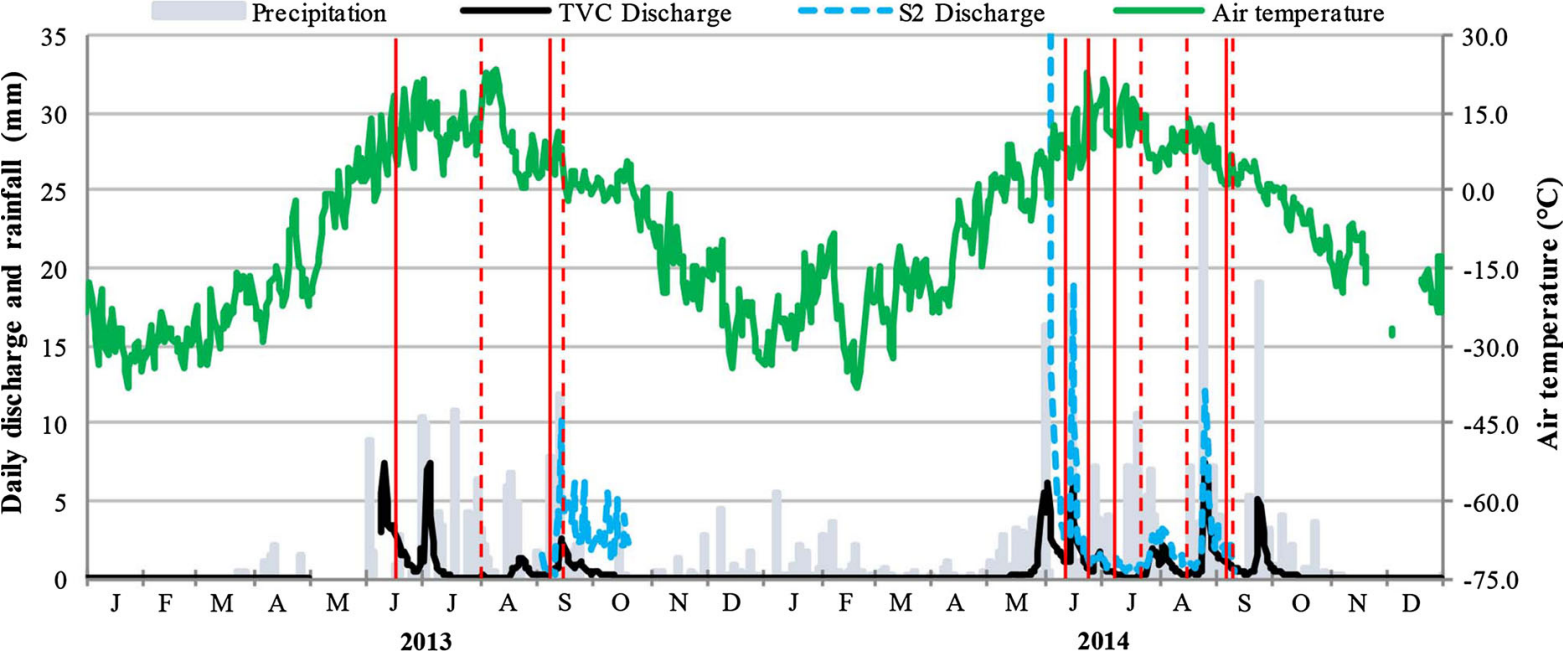
Daily discharge (Trail Valley Creek), precipitation and mean air temperature (Environment Canada meteorological station 220N005) during the sampling years 2013 and 2014; sampling dates are shown by the *vertical lines*, *solid lines *represent when all locations were sampled, and the* dashed lines* represent when only the Siksik catchment sites were sampled

The study area (68°44′54.5″N, 133°29′41.7″W) is located east of the Mackenzie River Delta, *c.* 45 km north of the town of Inuvik, Northwest Territories, Canada, and north of the shrub-tundra transition zone. The total study area covers approximately 14 km^2^, including the 0.94 km^2^ Siksik Creek catchment (Fig. 1), with an elevation range of approximately 50–110 m above sea level. Within this area are six lakes (ranging from 0.006 to 0.34 km^2^) among gently rolling hills with Siksik Creek draining into a larger stream system, Trail Valley Creek (Fig. 1). Trail Valley Creek, a 68.3 km^2^ catchment, flows east, through a relict glacial meltwater channel incised into the Arctic plateau and drains into the saline Husky Lakes, which are connected to the Beaufort Sea (Teare 1998). Climate in the study area and the discharge of Trail Valley Creek have been monitored by Environment Canada since 1977 (Endrizzi and Marsh 2010; Quinton and Marsh 1998, 1999; Quinton et al. 2000; Quinton and Pomeroy 2006); the Trail Valley Creek gauging station is just upstream of the Siksik confluence (Fig. 1). The discharge towards the bottom of Siksik Creek catchment was monitored during the 2013–2014 sampling seasons, but there was some equipment failure resulting in a patchy record, while the Trail Valley Creek discharge data was more consistently available (Fig. 2).

The study area is in the continuous permafrost zone of the western Canadian Arctic, with an average end of season active layer thickness (ALT) in Siksik catchment of *c.* 0.5 m in both 2013 and 2014 (see “Controls on aquatic geochemistry” section). Mean air temperatures were −24.9 °C for January to April and 6.7 °C for May to September in 2013, −16.7 °C for October 2013 to April 2014, and 7.1 °C for May to September 2014 (Fig. 2). These fall close to the mean monthly temperatures in the region of 7.7 °C (±1.1 σ) from May to September, and −20.9 °C (±2.1 σ) from October to April (Teare 1998; Environment Canada, accessed May 2015). However, the annual average air temperature in Inuvik has increased by 2.5 °C since 1970, and active layer depths at Illisarvik on Richards Island, *c.* 120 km north-northwest of the site, have increased by around 8 cm since 1983 (Burn and Kokelj 2009).

Precipitation during the study period was 162 mm in 2013 and 277 mm in 2014 (Fig. 2), bracketing mean annual precipitation from 1960 to 2005 (253 mm ± 49 σ, of which 38 % ± 18 σ fell as rainfall—note, however, that winter precipitation data in this area can be unreliable; Environment Canada, accessed May 2015; Marsh et al. 2000). The hydrology of the area is dominated by spring snowmelt (*c.* 90 % of annual flow; Quinton and Marsh 1999) following an eight-month snow covered season, while the growing season is limited to June through August (Quinton et al. 2000). The freshet commenced in late May (Fig. 2) with deep snow beds remaining until mid-June in both 2013 and 2014. Average daily streamflow during the growing season for 2013 and 2014 was 1.3 and 1.2 mm day^−1^, respectively, in Trail Valley Creek, and 2.3 mm day^−1^ in Siksik for 2014, however both miss the major freshet event (Fig. 2). Average annual streamflow in Trail Valley Creek for the same period (June to October) from 1979 to 2011 was 0.6 mm (±0.2 σ).

The vegetation of the area consists predominantly of ericaceous shrubs, sedges (*Eriophorum* and *Carex* spp.), bryophytes and lichens, with patches of tall shrubs on hillslopes (*Alnus viridis* and *Betula* spp.). The riparian areas are characterized by *Betula glandulosa* and *Salix* spp.).

### Geology

The underlying geology of the Siksik Creek catchment and surrounding lakes system is Miocene/Pliocene in age and comprises unconsolidated chert, quartzitic sandstone and siltstone alluvial gravel that makes up the Tertiary Beaufort Formation (Rampton 1987; Teare 1998). This is overlain by a 1-m thick Quaternary Pleistocene till, deposited following the early (*c.* 100,000 years BP) and late (*c.* 21,000–25,000 years BP) Wisconsin glaciation (Rampton 1987). The surface topography overlying this till layer is dominated by organic-rich topsoil, between 0.05 and 0.5 m thick, underlain by mineral soils in a heterogeneous hummock/inter-hummock morphology. The hummocks are on average 0.4–1 m wide and 0.1–0.4 m high, consisting of mainly mineral soils, while the inter-hummocks are filled with 0.2–0.5 m of loose peat, moss and vascular plant litter, which may contain 5–10 cm wide soil pipes; these are thought to be the primary controls of site hydrology (Quinton and Marsh 1998, 1999; Quinton et al. 2000; Quinton and Pomeroy 2006). The organic-rich peat layers consist of weak to strongly decomposed plant material, accumulated during the last *c.* 1,000 to *c.* 30,000 years (Rampton 1987). Physical weathering along the main branch of Trail Valley Creek has caused the deposition of some fine-silt to coarse-pebbled colluvial material within the system. Cryoturbation is common in the region, resulting in periglacial structures such as mud boils and ice-wedge polygons. There are relatively few thermokarst features in the study area; what few there are include thaw lakes and several active layer detachments (Rampton 1987; Quinton and Marsh 1998).

### Sampling strategy

Water was collected for analysis of bulk chemistry and dissolved C species from 27 different sites throughout the study area (Fig. 1). Sampling sites were selected to represent the contrasting types of surface water bodies and geomorphological settings that can be found in this part of the western Canadian Arctic tundra, including lakes, pools and streams of different size. The sampling sites were then grouped together into water types based on their hydrological and geomorphological setting: multiple lakes and their outlets, polygonal pools associated with ice-wedge polygons, and streams including Siksik Creek, lake inlets and Trail Valley Creek (a larger, 68.3 km^2^ catchment, regional stream system; Fig. 1).

These three geomorphologically distinct water types were sampled on 6–18 occasions during the snow-free seasons in 2013 and 2014 (Fig. 2). The lake samples (“Lake”) were taken from shore-side at the mid-point and outlet of six lakes (Fig. 1); the combined total area of these lakes was 0.73 km^2^. The polygonal pool samples (“Polygon”) were collected from pools ranging in surface area from 0.5 to 3.0 m^2^ that form in between the ice-wedges; samples were collected from three different polygonal areas totaling 0.04 km^2^, including polygons that feed Siksik Creek and a lake (Fig. 1). The stream samples (“Stream”) were collected from four points along the 3.1 km long Siksik Creek channel, three points along Trail Valley Creek immediately above and below the Siksik confluence, and five lake inlet locations (Fig. 1; note that these sites are part of a larger study, hence the reason why site names are not in strict numerical order).

## Methods

Sampling campaigns took place during the period between spring snowmelt (early June) and autumn (early September) of 2013 and 2014 (Fig. 2). Surface water samples (*n* = 238) were collected using a 60 ml syringe from approximately 5 cm depth in the water column and the sample injected through 0.45 µm Millipore syringe-driven filters and stored without headspace in 30 or 60 ml bottles that were first rinsed with the filtered sample. In situ field parameters (pH, electrical conductivity—EC, and temperature) were measured at the same time as sampling using Hanna Instruments^®^ HI-9033 and HI-9124 meters. Samples were kept cool (<6 °C) and dark in the field prior to refrigeration in the laboratory. The filtrate from one sample bottle was analysed for DOC and DIC at the Centre for Ecology and Hydrology (CEH—Edinburgh, UK) on a PPM LABTOC Analyser (detection range of 0.1–4000 mg l^−1^); concentrations were calculated based on a three point calibration curve with a maximum of 50 mg l^−1^. The filtrate from the second sample bottle was analysed for major ions at the University of Stirling (Stirling, UK) using a DX-120 IC (Dionex Corp.—Cl^−^, NO_3_^−^, and SO_4_^2−^; minimum detection limit of 0.03 mg l^−1^) and an iCAP 6000 ICP-OES (Thermo Fisher Scientific Inc.—Al^3+^, Ca^2+^, total Fe, K^+^, Mg^2+^, total Mn, Na^+^, P and Si; minimum detection limit of 0.05 mg l^−1^).

Dissolved CO_2_ and CH_4_ gas samples (*n* = 219) were collected using the headspace technique (Kling et al. 1992a; Dinsmore et al. 2013), where a 20 ml headspace of ambient air was equilibrated with a 40 ml water sample (collected from between 2 and 10 cm water depth) in a 60 ml syringe at natural stream temperature by shaking the syringe for 1 min vigorously underwater at the sampling point. Sampling depth was noted and used to calculate total (air plus water) system pressure. The equilibrated headspace (20 ml) was then injected into a pre-evacuated gas-tight borosilicate Exetainer^®^ tube (Labco, UK) and transported back to the UK. Multiple ambient samples were collected and stored in Exetainer^®^ tubes throughout each day’s sampling trip. Both headspace and ambient samples were analysed at CEH, Edinburgh on an HP5890 Series II gas chromatograph (Hewlett–Packard) with flame ionization detector and attached methaniser for CH_4_ and CO_2_, respectively. Detection limits for CO_2_ and CH_4_ were 7 ppmv and 84 ppbv, respectively. Concentrations of CO_2_ and CH_4_ dissolved in the stream water were calculated from the headspace and ambient concentrations using Henry’s Law (e.g. Hope et al. 1995).

Active layer depths were measured in June, July and September in both years at randomly located points along 3 transects crossing Siksik catchment (n = 10 points per transect). Depths were measured at 4 random spots within *c.* 1 m of each measurement point in both hummock and inter-hummock areas using a long steel rod marked with measurement graduations (i.e., n = 8 measurements per point, n = 30 points in total across the catchment).

Significant differences between the measured variables’ means for each group were analysed by applying an ANOVA in R (version 3.1.1) using a linear model, then testing the differences using the Tukey’s Honest Significant Differences test (P < 0.05; Table 1).

**Table 1:** Mean, standard error (SE) and coefficients of variation (CV) for aquatic chemistry of the three water types sampled in 2013 and 2014

	Lake (*n* = 57)		Polygon (*n* = 47)		Stream (*n* = 134)		All (*n* = 238)	
	Mean ± SE	CV	Mean ± SE	CV	Mean ± SE	CV	Mean ± SE	CV
pH	6.69 ± 0.05	0.05 ^a^	5.74 ± 0.15	0.16 ^b^	6.28 ± 0.04	0.07 ^c^	6.28 ± 0.05	0.10
Temperature (°C)	8.6 ± 0.6	0.52 ^a^	6.1 ± 0.4	0.45 ^b^	4.8 ± 0.3	0.75 ^b^	6.0 ± 0.3	0.67
EC (µS/cm)	63.7 ± 3.4	0.37 ^a^	57.3 ± 4.6	0.49 ^ab^	50.4 ± 2.0	0.36 ^b^	55.7 ± 1.7	0.41
DOC (mg C l^−1^)	16.6 ± 0.7	0.32 ^a^	32.6 ± 1.0	0.21 ^b^	23.0 ± 0.6	0.30 ^c^	23.4 ± 0.54	0.36
DIC (mg C l^−1^)	2.51 ± 0.28	0.82 ^a^	4.16 ± 0.53	0.86 ^b^	2.93 ± 0.20	0.79 ^b^	3.07 ± 0.17	0.85
CO_2_ (mg C l^−1^)	1.19 ± 0.10	0.62 ^a^	4.13 ± 0.42	0.66 ^b^	2.01 ± 0.10	0.56 ^c^	2.20 ± 0.12	0.81
CH_4_ (µg C l^−1^)	18.8 ± 3.4	1.35 ^a^	10.8 ± 5.4	3.17 ^ab^	4.71 ± 1.72	4.05 ^b^	9.43 ± 1.67	2.62
Al^3+^ (mg l^−1^)	BD		0.11 ± 0.05	0.94 ^a^	0.09 ± 0.05	0.74 ^a^	0.08 ± 0.05	0.94
Tot. Fe (mg l^−1^)	0.40 ± 0.05	0.72 ^a^	0.28 ± 0.05	0.65 ^b^	0.54 ± 0.05	0.68 ^b^	0.45 ± 0.05	0.74
Tot. Mn (mg l^−1^)	0.05 ± 0.05	1.37	BD		BD		BD	
Ca^2+^ (mg l^−1^)	6.58 ± 0.33	0.38 ^a^	6.89 ± 0.62	0.61 ^ab^	5.68 ± 0.20	0.41 ^b^	6.13 ± 0.19	0.46
K^+^ (mg l^−1^)	0.63 ± 0.05	0.45 ^a^	0.15 ± 0.05	1.45 ^b^	0.22 ± 0.05	1.01 ^b^	0.31 ± 0.05	0.98
Mg^2+^ (mg l^−1^)	2.70 ± 0.16	0.44 ^a^	3.16 ± 0.30	0.65 ^a^	2.69 ± 0.10	0.43 ^a^	2.79 ± 0.09	0.50
Na^+^ (mg l^−1^)	2.00 ± 0.15	0.56 ^a^	1.38 ± 0.09	0.42 ^b^	1.45 ± 0.05	0.41 ^b^	1.57 ± 0.05	0.50
Si (mg l^−1^)	1.04 ± 0.11	0.77 ^a^	2.79 ± 0.25	0.62 ^b^	2.84 ± 0.15	0.61 ^b^	2.40 ± 0.11	0.72
Cl^−^ (mg l^−1^)	1.66 ± 0.08	0.31 ^a^	1.16 ± 0.07	0.40 ^b^	1.18 ± 0.04	0.36 ^b^	1.30 ± 0.04	0.39
SO_4_^2−^–S (mg l^−1^)	3.85 ± 0.39	0.71 ^a^	0.73 ± 0.32	2.69 ^b^	1.82 ± 0.24	1.37 ^b^	2.11 ± 0.19	1.27

Different letters indicate significant differences (Tukey’s Honest Significant Difference, P < 0.05) between means within a variable. Note that *n* is the maximum number of observations for a given parameter, *n*_*min*_ ≥ 38 for all groups. BD = values below the minimum calibrated instrumental detection limit of 0.03–0.05 mg/l (see “Methods” section)

Principal components analysis (PCA) was carried out on the hydrochemical dataset to simplify the description of variability (Filella et al. 2014). Samples with missing values for the variables included in the PCA (DOC, DIC, CO_2_–C, CH_4_–C, Al^3+^, Ca^2+^, Cl^−^, total Fe, K^+^, Mg^2+^, total Mn, Na^+^, Si, and SO_4_^2−^–S), were first removed and the PCA was then applied to the remaining dataset (*n* = 181) using the “prcomp” function in R. The principal components (PCs) that best described the variability in the dataset were identified, and compared with the biogeochemical variables used to define the PCs (plus the descriptive variables pH, temperature, electrical conductivity, total dissolved solids—TDS, the sum of all measured chemical constituents of a water sample in mg l^−1^) using a Pearson pair-wise correlation (“rcorr” function, “Hmisc” package in R). This was done for the entire dataset, as well as the three water types individually.

To test if there was significant spatial connectivity between sites, Pearson pair-wise correlations (method as above) were carried out to determine “synchrony” between sites for each chemical species (after Kling et al. 2000). Aquatic synchrony analyses are intended to highlight the co-variance of geochemistry at sites that are well connected physically (e.g. stream sites upstream or downstream from one another), in comparison to those that are physically separated (e.g. two disconnected lakes). This was carried out for two groups; those sites outside Siksik catchment (sampled less regularly), and those within (sampled more regularly; Fig. 2). For each chemical species, correlation coefficients were determined for every possible pair of sites within a group, and the percentage of correlations that were significant (P < 0.05) was then calculated; we term this “spatial synchrony.” Conversely, to test for linkages between specific pairs of sites, the correlations were isolated by site and the average correlation was taken for all the C species and all the ions for each site pair (see “Statistical analysis” section); we term this “temporal synchrony.” Finally, we also analysed for “synchrony-through-time,” i.e. whether the variability of specific variables across all sites was correlated to the time of sampling. This was done using Pearson pair-wise correlations between each variable at each site and the day of year (DOY; i.e. day 1–365); this was also split based on whether sites were within or outside Siksik catchment. This method treats the sampling date as a variable, just like DOC concentration for example, and hence we test for whether a change in DOY is reflected in changes of chemical species concentrations. We further test for trends through time graphically, see “Controls on aquatic geochemistry” section.

To explore the potential influence of weathering inputs on dissolved C concentrations at the study site we examined the Ca^2+^ and Si ratios to Cl^−^. Si and Ca^2+^ are the most likely inputs from the underlying glacial geology, and their ratio to Cl^−^ accounts for the influence of element concentration due to evapotranspiration (Dean et al. 2014; Moulton et al. 2000). Cl^−^ at the study site is assumed to have a dominantly wet and dry airborne deposition source because the late Holocene marine transgression is unlikely to have reached the elevations of the study region (Hill et al. 1992; Campeau et al. 2000).

## Results

### Bulk chemistry

**Fig. 3:**
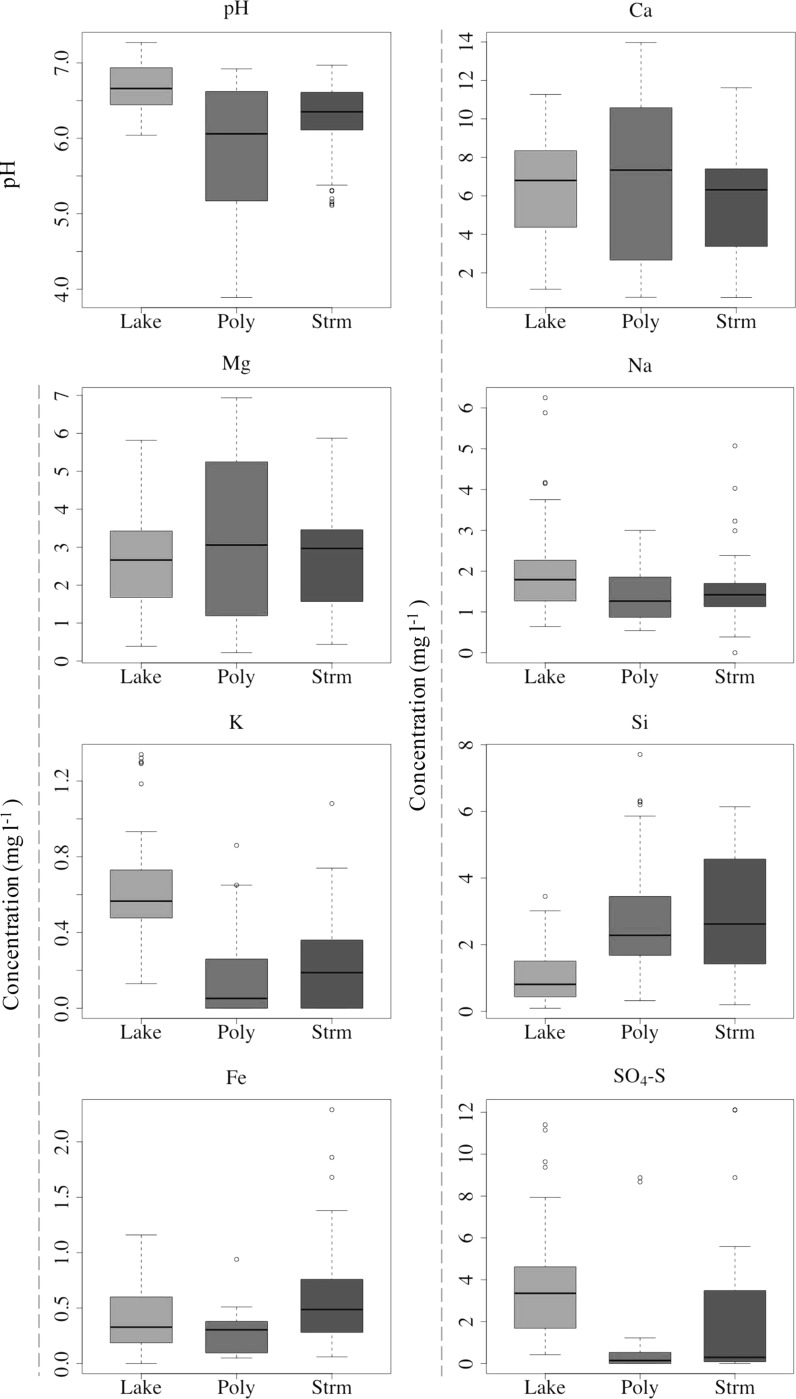
*Boxplots* of pH and primary ion concentrations in water samples collected during both the 2013 and 2014 field campaigns, grouped by water type (*Lake* Lake, *Poly* Polygon, *Strm* Stream). The thick *horizontal lines* represent the median, the *upper* and *lower* limits of the *boxes* represent the *upper* and *lower* quartiles, and the whiskers extend to 1.5 times the group’s interquartile range for the displayed variable; the *circles* represent outliers

Electrical conductivity (EC) was similar across the water types, with Stream having the lowest (50.4 ± 2.0 [standard error − SE] µS cm^−1^), and Lake the highest (63.7 ± 3.4 µS cm^−1^); variability was relatively low, with coefficients of variation (CVs) ranging from 0.36 to 0.49 (Table 1). Water temperature varied from 4.8 ± 0.3 to 8.6 ± 0.6 °C across the groups, with Stream the coldest and Lake the warmest, and CVs of 0.45 to 0.75 (Table 1). Mean pH of the water types (Table 1; Fig. 3) varied between 5.74 ± 0.15 and 6.69 ± 0.05 with Polygon samples the most acidic (5.74 ± 0.15), but most variable (CV = 0.16), and Lake the least acidic (6.69 ± 0.05).

Calcium (Ca^2+^) was the dominant cation in the waters (average 6.13 ± 0.19 mg l^−1^ across all samples), with the greatest variability observed in the Polygon samples (CV = 0.61; Fig. 3). Magnesium (Mg^2+^) was the next dominant cation (average 2.79 ± 0.09 mg l^−1^ across all samples), followed by sodium (Na^+^; average 1.57 ± 0.05 mg l^−1^ across all samples); both Mg^2+^ and Na^+^ had similar concentrations in all water types (CVs of *c*. 0.50 for both ions across all samples; Table 1). The Polygon samples again had the highest variability in Mg^2+^ (CV = 0.65), but the Lake samples had the most variability in Na^+^ concentrations (CV = 0.56; Fig. 3). Silica (Si) was lower in the Lake samples (1.04 ± 0.11 mg l^−1^) compared to Stream (2.84 ± 0.15 mg l^−1^) and Polygon samples (2.79 ± 0.25 mg l^−1^; Fig. 3). The dominant metal was (total) Fe (0.28 ± 0.03 to 0.54 ± 0.05 mg l^−1^), with lowest and highest concentrations in the Polygon and Stream samples, respectively (Fig. 3).

**Fig. 4:**
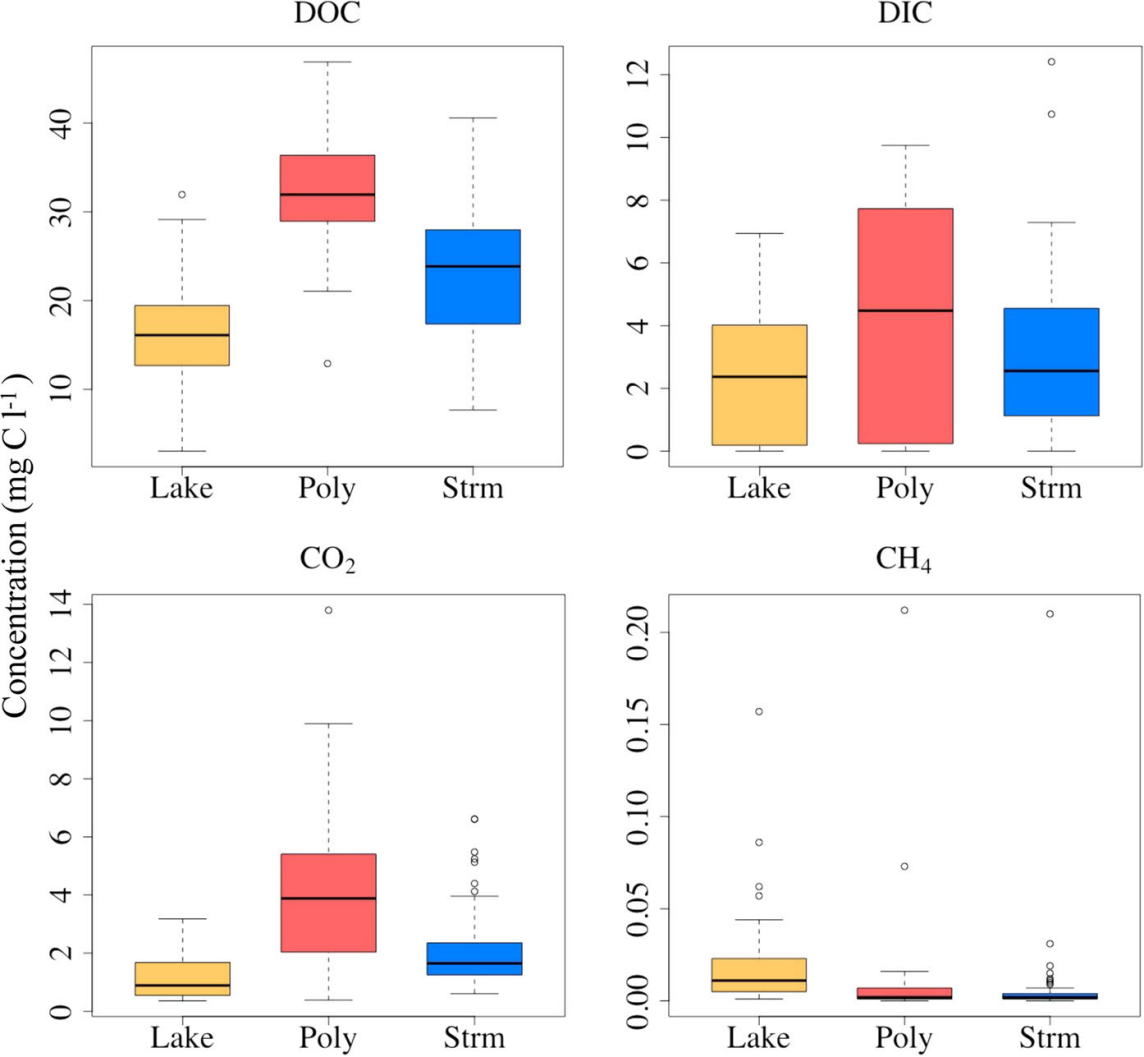
*Boxplots* of dissolved C species concentrations in water samples collected during both the 2013 and 2014 field campaigns, grouped by water type (see legend to Fig. 3); the format of the *boxplots* is also the same as in Fig. 3

Sulphate (SO_4_^2−^–S) was the dominant anion in the Lake samples (3.85 ± 0.39 mg l^−1^; Fig. 3), but was lower in Polygon (0.73 ± 0.32 mg l^−1^) and Stream (1.82 ± 0.24 mg l^−1^) samples, where DIC (a proxy for HCO_3_^−^, which was not measured directly in this system; see “Carbon species” section; Fig. 4) was the dominant anion.

Nitrate (NO_3_^−^–N) was not analysed in 2013, and was only detectable in one Lake sample in 2014 (0.26 mg l^−1^), so was not considered important in the aquatic system. Phosphorous (P) was only present in very low concentrations in a few samples, being generally below the detection limit. Potassium (K^+^), however, was present in most samples: higher in the Lake samples (0.63 ± 0.05 mg l^−1^) compared to the Polygon and Stream samples (0.15 ± 0.05 and 0.22 ± 0.05 mg l^−1^ respectively; Fig. 3).

### Carbon species

DOC concentrations were highest in the Polygon group (32.6 ± 1.0 mg C l^−1^), higher than both the Stream (23.0 ± 0.6 mg C l^−1^) and Lake (16.6 ± 0.7 mg C l^−1^). DOC concentrations did not vary substantially within the groups (CVs of 0.21–0.32; Table 1; Fig. 4).

DIC concentrations followed a similar pattern to DOC, with the highest concentrations observed in the Polygon (4.16 ± 0.53 mg C l^−1^), followed by the Stream (2.93 ± 0.20 mg C l^−1^) and Lake (2.51 ± 0.28 mg C l^−1^) groups. DIC variability was consistently greater than DOC, with CVs of 0.79 to 0.86 (Table 1; Fig. 4).

The highest CO_2_ concentrations were again in the Polygon group (4.13 ± 0.42 mg C l^−1^); the Stream and Lake concentrations were at least 50 % lower (2.01 ± 0.10 and 1.19 ± 0.10 mg C l^−1^, respectively); variability in CO_2_ concentration (CVs 0.56–0.62) was in between that of DIC and DOC (Fig. 4). CH_4_ concentrations in comparison were consistently low compared to the other C species across all groups (generally <0.05 mg C l^−1^; Table 1), but were highly variable (CVs of 1.35–4.05; Fig. 4).

**Fig. 5:**
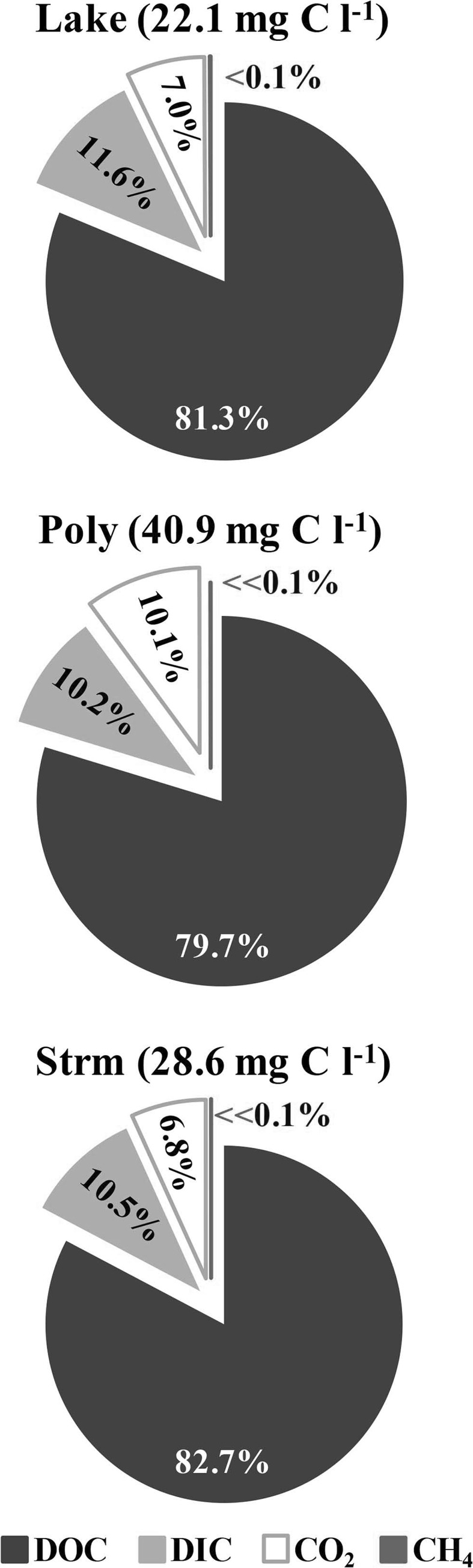
Exploded pie charts of the relative proportions of dissolved C species concentrations in water samples collected during both the 2013 and 2014 field campaigns, grouped by water type (see legend in Fig. 3). The average total C content of for each group is given next to the group labels; CH_4_ concentrations were <0.1 % of the total C content for all groups

Total C concentrations were highest in the Polygon group (40.9 mg C l^−1^), followed by Stream (27.9 mg C l^−1^) and Lake (20.3 mg C l^−1^; Fig. 5). The Polygon samples were characterised by higher concentrations of all C species compared to the other water types, with the exception of CH_4_, which was slightly higher in the Lake samples (Fig. 4). The relative proportion of the different C species to each other was similar across all water types (Fig. 5), although the total C concentrations varied significantly. DOC accounted for 80 % or more of total C concentrations, followed by DIC (*c.* 10–15 %) and CO_2_ (5–10 %); CH_4_ was less than 0.1 % (Fig. 5). DIC concentrations in the Polygon samples were comparable to CO_2_; in the other groups DIC was greater.

To explore the potential influence of weathering inputs on dissolved C concentrations at the study site we examined the relationship between Ca^2+^ and Si concentrations, and Ca^2+^/Cl^−^ and Si/Cl^−^ ratios with aquatic C species. The Ca^2+^ and Si concentrations, and Ca^2+^/Cl^−^ and Si/Cl^−^ ratios correlated poorly with DOC (maximum R^2^ < 0.08), DIC (maximum R^2^ < 0.05), CO_2_ (maximum R^2^ < 0.01) and CH_4_ concentrations (maximum R^2^ < 0.01).

### Statistical analysis

The differing geochemistry of the water types is described in the previous two sections, and is highlighted by the Tukey’s Honest Significant Differences test (Table 1). The mean values for the Polygon and Stream samples were significantly different for three variables (pH, DOC and CO_2_), contrasting with the mean values for the Lake and Polygon samples which were significantly different for all variables except EC, CH_4_, Ca^2+^ and Mg^2+^; Mg^2+^ was the only variable for which the means of all the water types were indistinguishable.

In order to simplify the variability inherent in the dataset, we first carried out principal components analysis (PCA) on the entire dataset. The first six principal components (PCs) each explained at least 5 % of the variability within the entire dataset (and also for each of the three water types), and cumulatively explained 79 %; all other PCs were omitted from further analysis. The PCs were then correlated with the measured variables (Table 2). PCs 1 and 2 described almost half the variability within the data and are both highly correlated with virtually all the variables in the dataset (Table 2). The rest of the PCs correlated with at least half of the variables, but only total Mn is significantly correlated with all PCs for the entire dataset.

**Table 2:** *P*-values from Pearson pair-wise correlation indicating which variables in the original dataset the principal components are correlated with

	PC1 (26 % var.)	PC2 (21 % var.)	PC3 (11 % var.)	PC4 (9 % var.)	PC5 (7 % var.)	PC6 (5 % var.)	Significance across groups
CO_2_–C	<0.001**	<0.001**	NS	<0.001**	NS	NS	12
DOC	<0.001**	<0.001**	NS	NS	0.026*	NS	11
CH_4_–C	NS	NS	NS	<0.001**	0.003*	<0.001**	10
Na^+^	<0.001**	0.013*	<0.001**	NS	<0.001**	0.025*	10
Tot. Fe	<0.001**	<0.001**	<0.001**	NS	<0.001**	NS	9
Al^3+^	<0.001**	0.012*	<0.001**	0.009*	NS	0.004*	8
Cl^−^	<0.001**	0.001*	<0.001**	NS	0.002*	NS	8
K^+^	<0.001**	<0.001**	NS	0.005*	<0.001**	NS	8
TIS	<0.001**	<0.001**	<0.001**	NS	0.004*	0.011*	7
DIC	NS	<0.001**	NS	0.001*	0.001*	<0.001**	7
Mg^2+^	<0.001**	<0.001**	NS	0.013*	<0.001**	NS	7
SO_4_^2−^–S	<0.001**	<0.001**	NS	NS	<0.001**	NS	7
Si	<0.001**	<0.001**	<0.001**	<0.001**	<0.001**	NS	7
pH	<0.001**	NS	0.008*	NS	NS	<0.001**	6
EC	<0.001**	0.027*	0.046*	0.001*	NS	<0.001**	6
TDS	<0.001**	0.001*	<0.001**	NS	<0.001**	NS	5
Ca^2+^	<0.001**	<0.001**	NS	NS	0.004*	NS	5
Temp.	<0.001**	NS	NS	0.020*	NS	0.025*	4
Tot. Mn	0.001*	<0.001**	<0.001**	0.023*	<0.001**	<0.001**	4

The percentages indicate the amount of variability within the entire combined dataset that the canonical accounts for. The “significance across groups” value is the summed occurrence of that variable as being significant to the first six PCs in PCAs carried out for each of the three water groups individually (out of a possible maximum of 18)

*NS* indicates values that are not significant, *EC* electrical conductivity, *TIS* total inorganic solids, *TDS* total dissolved solids

* Indicates values that are significantly correlated (*P* < 0.05); ** indicates values that are highly significantly correlated (*P* < 0.001)

PCAs were also carried out for the each of the individual water types. The first six PCs were again selected (describing cumulatively between 79 and 91 % of variability in each group), and the number of times a variable was correlated with a PC across all three of the water types was collated (Table 2). This demonstrates how often a variable was significant in describing the variability in each PC across the PCAs for each group, and therefore how important it was in defining the overall water geochemistry, in comparison with the overall PCA. In this case total Mn was the least significant, along with temperature, Ca^2+^, TDS, EC and pH (Table 2); the most consistently significant variables were CO_2_–C, DOC, CH_4_–C and Na^+^ (Table 2). This indicates that the latter variables are key to differentiating/grouping the water types by biogeochemical processes.

To determine the level of geochemical connectivity we estimated the spatial synchrony of chemical species across the different sampling points (Kling et al. 2000). The percentage of significant correlations (Table 3) shows, for each chemical species, how often the concentrations co-varied across the different sampling sites. Ca^2+^ and Mg^2+^, the major cations in the system (see “Bulk chemistry” section), were the most synchronised species across the sites outside Siksik catchment, along with TDS and Si (Table 3). In the sites within Siksik catchment Ca^2+^, Mg^2+^ and TDS were again highly synchronised, but Si and K^+^ were the most synchronised (Table 3).

**Table 3:** Spatial synchrony analysis results: the average relationship between site pairs (Pearson’s *r* values) outside and within the Siksik catchment (Fig. 1) using Pearson correlations, and the proportion of those relationships that are significant (P < 0.05; after Kling et al. 2000)

Sites outside Siksik catchment			Sites within Siksik catchment		
Variable	Pearson’s *r*	Significant correlations (%)	Variable	Pearson’s *r*	Significant correlations (%)
Ca^2+^	0.75	70.6	Si	0.90	91.7
Mg^2+^	0.76	65.4	K^+^	0.80	80.6
TDS	0.77	53.7	Ca^2+^	0.67	66.7
Si	0.81	50.0	Mg^2+^	0.68	66.7
SO_4_^2−^–S	0.73	41.9	TDS	0.67	63.9
Na^+^	0.68	41.2	DOC	0.38	41.7
Al^3+^	0.51	21.3	Al^3+^	0.37	41.7
Tot. Mn	0.21	19.9	Na^+^	0.49	38.9
CO_2_–C	0.31	19.1	SO_4_^2−^–S	0.44	38.9
DIC	0.29	18.5	Cl^−^	0.21	27.8
pH	0.49	18.4	DIC	0.40	22.2
K^+^	0.15	16.2	CO_2_–C	0.43	22.2
Cl^−^	0.44	15.4	CH_4_–C	0.29	19.4
CH_4_–C	0.29	14.0	Tot. Mn	0.03	19.4
DOC	0.39	12.5	Tot. Fe	0.19	11.1
Tot. Fe	0.00	12.5	pH	0.29	5.6

The temporal synchrony between specific site pairs (all possible pairs of sites either within or outside Siksik catchment; Fig. 1) is shown in Tables 4 and 5. The mean synchrony in the sites outside Siksik catchment is 0.34 ± 0.25 for the C species, and 0.52 ± 0.19 for the ions (error ranges are one standard deviation; Table 4). The mean synchrony in the sites within Siksik catchment is 0.38 ± 0.18 for the C species, and 0.48 ± 0.14 for the ions (Table 5).

**Table 4:** Temporal synchrony analysis results: the relationship between the chemistry of site pairs outside the Siksik catchment (Fig. 1) using Pearson correlations

	L1	L2	L3	L4	L5	L6	L7	L8	L9	L10	L11	L12	L13	L14	L15	L16	L18
L1		**0.42**	**0.48**	**0.39**	**0.43**	**−0.17**	**−0.30**	**0.25**	**0.26**	**0.29**	**0.34**	**0.21**	**0.39**	**0.18**	**0.25**	**0.48**	**0.35**
L2	0.88		**0.21**	**0.62**	**0.42**	**0.00**	**0.03**	**0.01**	**0.34**	**0.15**	**0.10**	**0.10**	**0.17**	**0.27**	**0.42**	**0.25**	**0.31**
L3	0.58	0.37		**0.51**	**0.40**	**0.26**	**−0.07**	**0.09**	**0.65**	**0.52**	**0.43**	**0.73**	**0.77**	**0.45**	**0.52**	**0.92**	**0.32**
L4	0.61	0.57	0.37		**0.51**	**0.37**	**0.31**	**0.39**	**0.61**	**0.56**	**0.12**	**0.31**	**0.41**	**0.57**	**0.76**	**0.61**	**0.39**
L5	0.36	0.38	0.57	0.24		**0.38**	**0.35**	**0.11**	**−0.18**	**0.18**	**−0.16**	**0.15**	**−0.19**	**−0.17**	**−0.20**	**−0.12**	**0.36**
L6	0.43	0.43	0.38	0.46	0.27		**0.36**	**0.44**	**0.12**	**0.43**	**−0.28**	**0.15**	**0.17**	**0.30**	**0.17**	**0.23**	**0.33**
L7	0.56	0.61	0.43	0.36	0.61	0.52		***0.25***	***0.26***	***0.38***	***−0.17***	***−0.06***	***0.00***	***0.07***	***0.17***	**−0.06**	**0.28**
L8	0.14	0.01	0.61	0.18	0.59	−0.14	*0.39*		***0.54***	***0.78***	***0.41***	***0.56***	***0.73***	***0.64***	***0.49***	**0.87**	**0.56**
L9	0.54	0.31	0.01	0.59	0.34	0.46	*0.40*	*0.76*		***0.65***	***0.19***	***0.56***	***0.65***	***0.45***	***0.49***	**0.69**	**0.38**
L10	0.60	0.50	0.31	0.71	0.35	0.39	*0.53*	*0.68*	*0.69*		***−0.34***	***0.41***	***0.48***	***0.27***	***0.36***	**0.53**	**0.51**
L11	0.67	0.52	0.50	0.42	0.38	0.52	*0.61*	*0.29*	*0.73*	*0.55*		***0.57***	***0.39***	***0.51***	***0.30***	**0.48**	**0.20**
L12	0.63	0.42	0.52	0.41	0.38	0.54	*0.51*	*0.34*	*0.78*	*0.58*	*0.92*		***0.75***	***0.53***	***0.52***	**0.75**	**0.36**
L13	0.58	0.48	0.42	0.43	0.33	0.56	*0.48*	*0.27*	*0.76*	*0.56*	*0.80*	*0.86*		***0.44***	***0.49***	**0.81**	**0.07**
L14	0.50	0.38	0.48	0.44	0.50	0.40	*0.54*	*0.52*	*0.75*	*0.59*	*0.79*	*0.78*	*0.71*		***0.74***	**0.49**	**0.24**
L15	0.57	0.34	0.38	0.53	0.39	0.46	*0.50*	*0.33*	*0.81*	*0.63*	*0.81*	*0.86*	*0.74*	*0.83*		**0.53**	**0.24**
L16	0.53	0.40	0.34	0.51	0.27	0.61	0.46	−0.02	0.79	0.60	0.73	0.77	0.77	0.64	0.75		**0.35**
L18	0.63	0.60	0.40	0.45	0.48	0.48	0.73	0.44	0.48	0.51	0.54	0.53	0.41	0.44	0.51	0.48	

Pearson’s *r* values for C species are shown in the upper right of the table (in bold), and inorganic solutes in the lower left. L7–15 values in italics—these sites appear to be well connected in the satellite image (Fig. 1; see “Controls on aquatic geochemistry” section). Mean for C species = 0.34 ± 0.25 (1σ); mean for inorganic solutes = 0.52 ± 0.19 (1σ)

**Table 5:** Temporal synchrony analysis results: the relationship between the chemistry of site pairs within the Siksik catchment (Fig. 1; L18 was excluded as there were not enough data points) using Pearson correlations. Pearson’s *r* values for C species are shown in the upper right of the table (in bold), and inorganic solutes in the lower left

	S1	S4	S5	S8	S10	T1	T2	L17	L19
S1		**0.42**	**0.72**	**0.29**	**0.02**	**0.57**	**0.50**	**0.17**	**0.36**
S4	0.62		**0.54**	**0.62**	**0.20**	**0.34**	**0.35**	**0.42**	**0.16**
S5	0.64	0.58		**0.47**	**0.17**	**0.59**	**0.52**	**0.34**	**0.66**
S8	0.34	0.30	0.49		**0.35**	**0.44**	**0.54**	**0.47**	**0.22**
S10	0.33	0.33	0.37	0.41		**0.21**	**0.14**	**0.01**	**0.28**
T1	0.57	0.35	0.51	0.34	0.28		**0.73**	**0.37**	**0.37**
T2	0.58	0.39	0.47	0.33	0.29	0.83		**0.45**	**0.27**
L17	0.58	0.52	0.66	0.50	0.42	0.35	0.42		**0.24**
L19	0.71	0.48	0.71	0.39	0.43	0.47	0.57	0.65	

Mean for C species = 0.38 ± 0.18 (1σ); mean for inorganic solutes = 0.48 ± 0.14 (1σ)

Synchrony-through-time is more important for the ions compared to the C species for the sites within as well as outside Siksik catchment (Table 6). For sites within Siksik catchment, Si, K^+^, TDS, Ca^2+^, Mg^2+^, SO_4_^2−^–S and Cl^−^ were all significantly correlated with the sampling day of year for the majority of samples. For the sites outside Siksik catchment, Na^+^, SO_4_^2−^–S, Ca^2+^, Mg^2+^ and TDS were also correlated with sampling day. The C-species in both site groups were only significantly correlated with the sampling day of year for less than 30 % of samples (Table 6).

**Table 6:** Synchrony-through-time results: the average relationship (Pearson’s *r* values) between site variables and the time of sampling (day of year) outside and within the Siksik catchment (Fig. 1) using Pearson correlations, and the proportion of those relationships that are significant (P < 0.05)

Sites outside Siksik catchment			Sites within Siksik catchment		
Variable	Pearson’s *r*	Significant correlations (%)	Variable	Pearson’s *r*	Significant correlations (%)
Na^+^	0.82	68.8	Si	0.79	100.0
SO_4_^2−^–S	0.84	62.5	K^+^	−0.72	88.9
Ca^2+^	0.75	56.3	TDS	0.67	88.9
Mg^2+^	0.76	56.3	Ca^2+^	0.64	77.8
TDS	0.77	56.3	Mg^2+^	0.69	77.8
K^+^	−0.13	43.8	SO_4_^2−^–S	−0.23	66.7
Si	0.76	37.5	Cl^−^	0.61	66.7
CO_2_–C	−0.45	31.3	Al^3+^	0.33	44.4
DIC	−0.49	25.0	Na^+^	0.30	33.3
CH_4_–C	0.29	18.8	DOC	0.39	33.3
Cl^−^	−0.51	18.8	Tot. Fe	0.18	22.2
DOC	0.66	18.8	pH	−0.05	22.2
Tot. Fe	0.35	12.5	DIC	0.38	22.2
Al^3+^	0.05	12.5	CO_2_–C	0.15	0.0
pH	−0.10	6.3	CH_4_–C	−0.04	0.0

## Discussion

This study aimed to describe the geochemical signatures of the study waters across both space and time, and in turn identify the key biogeochemical controls on the aquatic chemistry at the study site. This is firstly addressed from the perspective of the geochemistry and then secondly, aquatic C concentrations.

### Controls on aquatic geochemistry

**Fig. 6:**
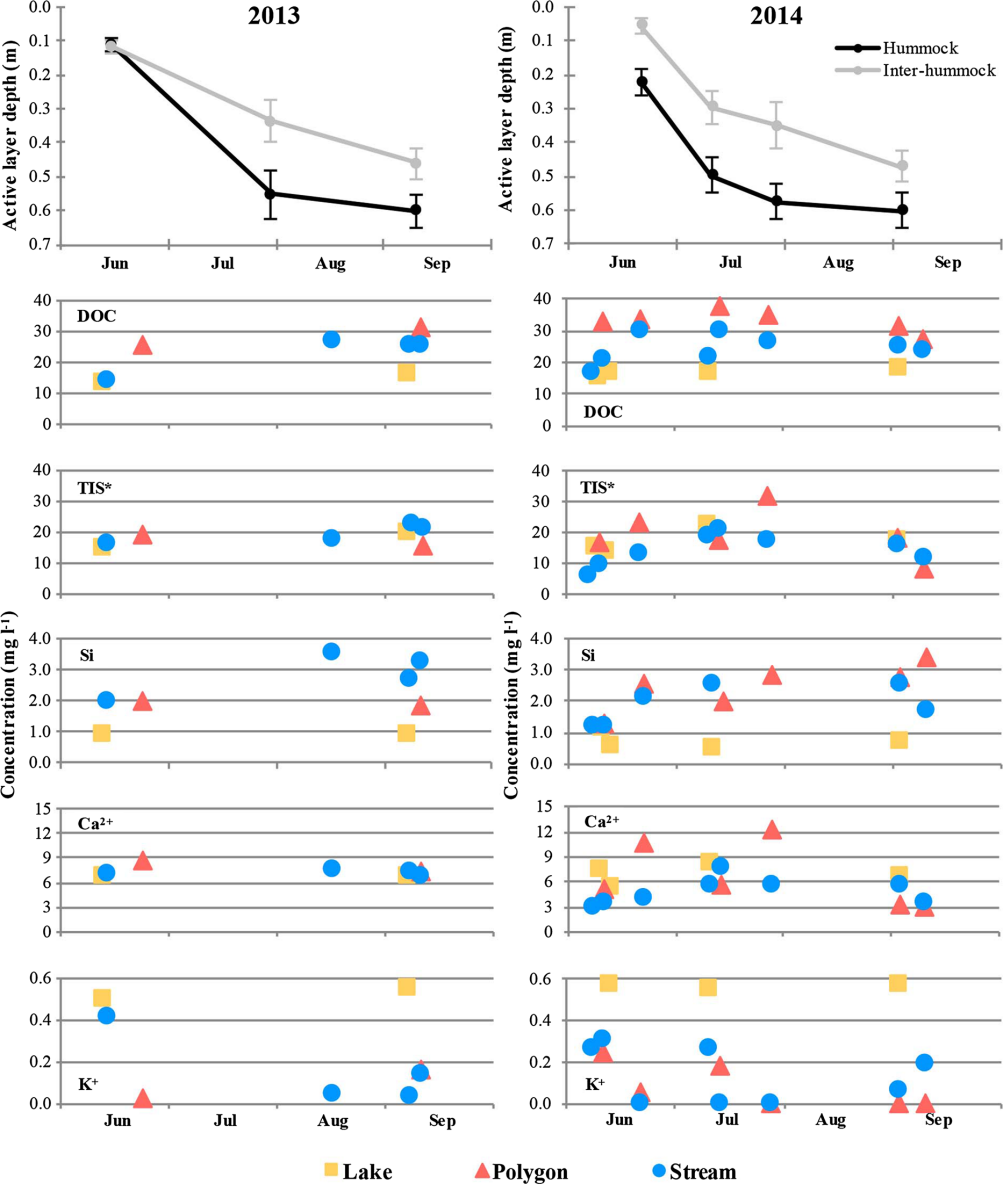
Seasonal active layer depth in hummock and inter-hummock areas (*top* figures) and selected species mean concentrations for each sampling period (*bottom ten* figures; see Fig. 2) for the 2013 and 2014 snow-free seasons (*left* and *right* figures, respectively); the x-axes are the same for each figure, only the year differs. *TIS is the sum of total inorganic solutes in the samples (Ca^2+^, K^+^, Mg^2+^, Si, Cl^−^, DIC [HCO_3_^−^], SO_4_^2−^; Frey and McClelland 2009)

The neutral to acidic pH across all groups (7.27–3.89; Fig. 3) likely reflects its origin in the organic-rich, upper soil layer of the study region through which much of the soil water flows due to the shallow active layer (Quinton and Pomeroy 2006; Fig. 6). This acidifies the water to a degree similar to that observed in temperate peatlands (pH 4.5–7.0; Billett and Moore 2007; Billett et al. 2007) and organic rich Western Siberian lowlands (pH 3.0–7.0; Shirokova et al. 2013), as opposed to the more buffered waters observed in the Alaskan, Eastern Canadian, Far East Siberian and Svalbard Arctic regions (pH 6.5–9.0; Crawford et al. 2013; Kling et al. 1992b; Mann et al. 2012; Negandhi et al. 2013, 2014; Stutter and Billett 2003). The slightly higher pH in the Stream and Lake groups relative to the polygons may indicate a lesser influence of this organic layer, or CO_2_ degassing (evasion) losses when soil water enters the lakes and streams, or even a greater carbonate input; however, Ca^2+^ concentrations in these groups do not support the latter conclusion (Fig. 3).

Electrical conductivity in the sampled waters was consistently low across all water samples (55.7 ± 1.7 µS cm^−1^; Table 1), in the lower range of reported values from other Alaskan, Canadian and Siberian Arctic sites (7–843 µS cm^−1^; Kling et al. 1992b; Kokelj et al. 2005; Mann et al. 2012; Shirokova et al. 2013). The low conductivity and pH of the sampled waters could also indicate that precipitation is important in defining the water chemistry, as rainfall in the region generally has a pH of *c.* 5.5 (Kling et al. 1992b) and a low conductivity of between 2.9 µS cm^−1^ (Kling et al. 1992b) and *c.* 8 µS cm^−1^ (median of 5 samples, unpublished data). However, the PCA highlights DOC and CO_2_ as significant in defining the different water groups’ biogeochemical signatures (Table 2), neither of which are likely to be derived from precipitation. The relatively high spatial synchrony of TDS (for which electrical conductivity is a proxy; Table 3) in the sites outside Siksik catchment may indicate that evapotranspiration plays an important role in defining the freshwater biogeochemistry. However, TDS was also significant in the sites within Siksik catchment where evapotranspiration is unlikely to be a controlling factor given the lower surface water area, unless the concentration of solutes by evapotranspiration is occurring primarily in the soil solution prior to entering the stream. This confirms that, despite precipitation being the principal direct source of surface water in the study area (deep groundwater contributions are negligible due to the shallow active layer), localised soil, fluvial and lacustrine processes are more important in defining the water chemistry (Quinton and Pomeroy 2006).

Previous studies have used water chemistry as an indicator of enhanced permafrost thaw and thermokarst processes (e.g. TIS, the total concentration of inorganic solutes; Frey and McClelland 2009; Kokelj et al. 2005). Degrading permafrost is expected to increase the concentration of inorganic solutes as subsurface water flow is able to interact with, and weather, deeper, newly thawed mineral soils and sediments (Kokelj et al. 2005). Depending on the geochemical composition of the frozen material, different chemical species may be leached into the aquatic system during thawing, such as Ca^2+^, K^+^, Mg^2+^, Na^+^, S and Si (Kling et al. 1992b; Kokelj et al. 2005; Stutter and Billett 2003; Frey et al. 2007; Keller et al. 2007; Frey and McClelland 2009). The synchrony-through-time analysis suggests that the time of sampling is important for sample biogeochemistry. Figure 6 explores this further, showing the time series of key chemical species identified in the PCA and synchrony analyses through the thaw seasons of both 2013 and 2014, along with the active layer depth. While there is some indication of an increase in TIS during 2013 (alongside DOC, Si and K^+^), this was not seen in 2014 where larger fluctuations than the 2013 seasonal increase were seen on much shorter time scales (Fig. 6). In 2014 in general, where the temporal resolution is higher, this increasing trend is only apparent at the first few sampling points of the year, with concentrations dropping towards the end of the measurement period (Fig. 6). This highlights the need for high resolution sampling when seeking to identify geochemical signatures in streams in these environments.

Ca^2+^ and Si are the most likely elements to be derived from mineral weathering of thawing permafrost material in the study area, particularly the addition of Ca^2+^ from calcareous material in parts of the system (Quinton and Pomeroy 2006). Ca^2+^ and Mg^2+^ were well correlated (R^2^ = 0.91), were the dominant cations in the system, and were both significant in the spatial synchrony analysis with positive Pearson r values (0.67–0.76), suggesting that these ions behave relatively conservatively within the system (Table 3). Furthermore, there is no relationship between Ca^2+^/Cl^−^ ratios and Cl^−^ (R^2^ = 0.27) which would indicate the introduction of Ca^2+^ to the system relative to (Cl^−^) inputs from wet and dry deposition. Ca^2+^ (and Mg^2+^) does not show a seasonal trend in either 2013 or 2014 (Fig. 6), indicating that the concentrations of these ions were not controlled by increasing weathering inputs as the active layer deepened through the thaw season. Ca^2+^ and Mg^2+^ are therefore important to the freshwater geochemistry of the site, but more likely derived from rainfall and/or dry deposition rather than weathering.

SO_4_^2−^–S was significantly higher in the lakes than in the Polygon and Stream samples (Table 1), although there was considerable variability in the latter two (Fig. 3). Increased SO_4_^2−^–S concentrations have been linked to permafrost thaw (Frey and McClelland 2009), suggesting that there may be more thawing in the lakes, releasing SO_4_^2−^, compared to the stream and polygon samples. However, such responses are not consistent (e.g. Parham et al. 2013), and the values measured here (0.73 ± 0.32 to 3.85 ± 0.39 mg l^−1^) are not especially high compared to similar systems in Alaska and Siberia (0.03 to 2.58 mg l^−1^; Petrone et al. 2006; Parham et al. 2013). The lower SO_4_^2^–S in the polygons and stream maybe also be due to increased sulphate reduction, linked to methane oxidation, in these waters or associated sediments, and this may explain the low CH_4_ concentrations in these samples (Fig. 4).

There are higher Si concentrations in the Polygon and Stream samples compared to the Lake samples, and this may be due to weathering inputs from the mineral soils in the region. Si/Cl^−^ ratios showed a slight relationship with Cl^−^ (R^2^ = 0.46), suggesting that inputs of Si from weathering (altering its relationship to the conservative ion, Cl^−^) may be an important process across the study area. Si is strongly correlated with the sampling day of year, particularly in sites within Siksik catchment, suggesting that it behaved conservatively in both site groups (Pearson’s r of 0.76 and 0.79 for sites outside and within Siksik catchment, respectively; Table 6). However, like Ca^2+^, an increasing seasonal trend is not evident in Fig. 6. The lower Si concentrations in the Lake samples compared to the Polygon and Stream samples are more likely explained by assimilation of Si by diatoms in the lake waters (Conley 2002), rather than greater weathering inputs of Si (relative to other ions) within the Siksik catchment.

The system presented here is relatively undisturbed, physically, compared to other parts of the Arctic, with relatively little visible evidence of recent thermokarst features and enhanced permafrost thaw, over and above seasonal thawing, with the exception of some few thaw lakes and several active layer detachments. At Toolik Lake in Alaska, for example, there is considerable evidence of enhanced permafrost thaw, and as a result increased contributions of inorganic solutes to the aquatic system have been reported (Hobbie et al. 1999). N, P and K^+^ are often the most noticeable elements derived from this increase in thawing, as Arctic regions are generally nutrient poor (Keller et al. 2007), and this was also observed at Toolik Lake (Bowden et al. 2012). Dissolved N_2_O and NO_3_^−^ were generally undetectable in this study; P was also generally undetectable in the water samples from our site, comparable to low concentrations observed at Toolik Lake (Kling et al. 1992b). Potassium (K^+^) was present at our study sites in low concentrations (0.31 ± 0.05 mg l^−1^; Fig. 2), overlapping the low end of concentrations at Toolik Lake (0.01–3.0 mg l^−1^; Kling et al. 1992b; Keller et al. 2007). Potassium was particularly low in the Polygon (0.15 ± 0.05 mg l^−1^) and Stream (0.22 ± 0.05 mg l^−1^) samples compared to the Lake group (0.63 ± 0.05 mg l^−1^; Fig. 3). This contradicts what might be expected, given that the Polygon samples are from the pools located between permafrost ice-wedges where the water has the greatest chance to interact directly with thawing permafrost, and that much of the Stream site waters originate from polygons (Fig. 1). K^+^ did correlate significantly in the synchrony-through-time analysis at sites within Siksik catchment, with a negative trend in time (Pearson’s r = −0.72; Table 6), but showed clear spatial synchrony with a positive spatial trend (Pearson’s r = 0.80; Table 3). This could suggest that, as the growing season progresses, K^+^ is taken up by plants, but also that K^+^ may be added to the stream from the decay of organic matter, possibly resulting from enhanced permafrost thaw. This pattern is not clear in Fig. 6, however, and is hard to separate from the overall variability at the site.

### Aquatic carbon biogeochemical controls

Rates of permafrost thaw are important in controlling DOC and DIC concentrations in Arctic streams and lakes (Olefeldt and Roulet 2014; Abbott et al. 2015). Aquatic DOC concentrations in this study ranged widely from 3.04 to 46.9 mg C l^−1^ across all groups (mean = 23.4 ± 0.54 mg C l^−1^; Table 1), falling in the mid-range of 2–55 mg C l^−1^ observed elsewhere in Arctic and sub-Arctic catchments (Dornblaser and Striegl 2015; Mann et al. 2012; Olefeldt and Roulet 2012; Olefeldt et al. 2014; Petrone et al. 2006; Prokushkin et al. 2011; Striegl et al. 2005). Areas undergoing elevated permafrost thaw have recorded values as high as 164 mg C l^−1^ (Shirokova et al. 2013), although thermokarst processes do not necessarily result in increased DOC concentrations in the current study region (Kokelj et al. 2005). Conversely, DIC in the study waters (0.00 to 12.4 mg C 1^−1^, mean = 3.07 ± 0.17) was predominantly at the lower end of the range observed elsewhere in the Arctic (generally 2–50 mg C l^−1^, using HCO_3_^−^ as a proxy for DIC where DIC values were not given; Dornblaser and Striegl 2015; Parham et al. 2013; Prokushkin et al. 2011; Tank et al. 2012), although some sites had much higher concentrations (50 to 391 mg C l^−1^; Kling et al. 1992a, b; Striegl et al. 2007), especially where carbonate was present in soil profiles (e.g. Frey et al. 2007). The low DIC concentrations in the waters presented here suggest that there is minimal input from carbonate weathering at the study site.

Active layer deepening and thermokarst features can increase DOC and CO_2_ concentrations when the active layer is shallow, channelling water through organic layers, and increase DIC concentrations where the active layer is deep enough to allow water to pass through mineral soils (Abbott et al. 2015; Dornblaser and Striegl 2015). DOC concentrations are consistently eight times greater than DIC (and CO_2_) in all water samples (Fig. 5), suggesting that flow paths were predominantly focused in the upper organic layer, rather than the mineral soils below. DOC concentrations did not show a significant trend through time (Fig. 6), indicating these flow paths did not alter much through the study seasons. DOC concentrations were highest in the Polygon group (Fig. 4), indicating that DOC contributions to the water in the ice-wedge pools were higher than in the other water types as a result of the longer residence times of these waters in the organic-rich layer and/or increased contact times with organic pool margins.

Dissolved CO_2_ concentrations ranged from 0.36 to 23.8 mg C l^−1^ (mean = 2.20 ± 0.12 mg C l^−1^; Fig. 4), comparable to 0.1 to 6.8 mg C l^−1^ for other Arctic aquatic studies (Crawford et al. 2013; Kling et al. 1992a, b; Negandhi et al. 2013; 2014; Shirokova et al. 2013; Striegl et al. 2012). The lower values of the Lake and Stream samples were similar to other stream and river values (0.1–2.0 mg C l^−1^; Crawford et al. 2013; Kling et al. 1992a; Striegl et al. 2012), and the Polygon samples more closely match values from other Arctic polygonal ponds and thermokarst features (0.4–6.8 mg C l^−1^; Negandhi et al. 2013, 2014; Shirokova et al. 2013). The highest CO_2_ concentrations observed in the Polygon samples were possibly due to increased contributions from/interactions with the soil zone, as observed in the DOC concentrations in the same waters.

Dissolved CH_4_ is present only in very low concentrations in the study waters, generally below 0.05 mg C l^−1^ (Fig. 4), similar to the 0.0002–0.21 mg C l^−1^ range observed in the Siberian Arctic (Shirokova et al. 2013). Although CH_4_ is generally below 0.1 % of total carbon concentrations in the sampled waters (Fig. 5) and is not considered an important form of aquatic C in this system, this could partly be due to the rapid transfer of this gas to the atmosphere, meaning that aquatic CH_4_ may be more important in the study area than these observations suggest. Evasion rates were not assessed in this study. In addition, the low CH_4_ values in the stream and polygon samples could also be related to sulphate reduction (see “Controls on aquatic geochemistry” section). Much of the CH_4_ release in Arctic aquatic environments is thought to occur through ebullition from the streambed and lake sediments (Walter et al. 2006; Tan et al. 2015), which was not measured in this study. It should also be noted that direct measurements of CH_4_ concentrations in soils and sediments across a transect from the Siksik Creek streambed upslope towards the edge of an alder thicket showed potentially very high in situ values (up to 16 % CH_4_) in 2014 (Street et al. accepted).

All C species were also poorly correlated with Ca^2+^/Cl^−^ and Si/Cl^−^ ratios, with R^2^ values all below 0.8 (see “Carbon species” section). A stronger correlation with DIC in particular would be expected if the mineral soils were markedly contributing to DIC concentrations; this was not evident.

### Biogeochemical implications

The C species CO_2_, DIC and DOC were consistently significant in defining the variability in the data set, along with Na^+^, Cl^−^ and total Fe (Table 2). This suggests that C concentrations were important for defining the geochemical variability in the system along with a minority of the inorganic solutes. Given that the total C concentrations in the samples (20.3–40.9 mg l^−1^; Fig. 5) were equal to or greater than TDS (21.3–27.1 mg l^−1^), they therefore comprise a key characteristic of the study site’s freshwater biogeochemistry.

This is supported by the synchrony analysis, where the mean temporal synchrony values of all the specific site pairs were lower for C species than for the inorganic solutes (both within and outside Siksik catchment; Tables 4 and 5), showing that C species are less well conserved in the system than the inorganic solutes. When we look specifically at the lake sites that appear to be physically well connected (L7 to L15; Fig. 1) the mean synchrony for these sites is 0.40 ± 0.26 and 0.63 ± 0.18 for the C species and ions, respectively (Table 4). These values are slightly higher than the subset (sites outside Siksik catchment) mean, but are within one standard deviation (Table 4). This again indicates that despite extracting sites that appear to be well connected spatially, there is less conservation of C species than the inorganic solutes; this suggests that many neighbouring sites are not well connected. The synchrony-through-time analysis further highlights the differences between the ions and the C species’ synchrony, with the most significant correlations with sampling day of year occurring primarily with the inorganic solutes, showing that that these species are more synchronous (i.e. co-vary more) in time than the C species (Table 6). This could be because of the conservative nature of the inorganic solutes in the site waters, and/or because of the spatially heterogeneous operation of processes such as degassing of gaseous C species (Raymond et al. 2013), or the microbial and photochemical degradation of DOC leading to degassing of its breakdown products (Vonk et al. 2015b).

The apparent conservative nature of the inorganic solutes as highlighted by the synchrony analyses, along with the lack of evidence of enhanced deepening of the active layer during the study period, suggests that the organic-rich inter-hummocks currently dominate the biogeochemistry of all the waters studied here. This shows that the importance of the hummock/inter-hummock topography to the Siksik hydrology, as identified by Quinton and Pomeroy (2006), also extends to the lakes.

Thawing permafrost can increase nutrient concentrations (N, P and K; Keller et al. 2007), and the “priming” ability of these nutrients may enhance allochthonous DOC and DIC respiration, and subsequent CO_2_ and CH_4_ production (Bianchi 2011; Marcé et al. 2015). Priming may render less labile organic C vulnerable to mineralisation if it is circulated through multiple lake systems and subjected to photo-oxidation and microbial respiration (Bianchi 2011; Cory et al. 2014). In our study, the relative proportion of different C species to each other remained consistent throughout the different water types (Fig. 5). There are, however, much lower total C concentrations in the Stream and Lake samples compared to the Polygon samples, and it is therefore possible that C is being sequestered, via plant uptake and/or burial in sediments, in the lakes (Walter Anthony et al. 2014) and stream beds, and subsequently released by CH_4_ ebullition (Walter et al. 2006; Street et al. accepted), which was not measured in this study. Alternatively, DOC may be lost in the Lake and Stream waters via microbial respiration (Spencer et al. 2015) and photo-oxidation (Cory et al. 2014), which is rapidly degassed to the atmosphere (Billett and Moore 2007; Long et al. 2015; Raymond et al. 2013), or diluted by water from other sources (e.g. precipitation, runoff and snowmelt). The vulnerability of DOC to mineralisation to CO_2_ means that pristine catchments with large DOC loads have the potential to be high CO_2_ sources. Whether one or a combination of these processes is occurring in these systems needs to be explored further using a C mass balance approach in order to contextualise the results presented here.

## Conclusions

The waters in this study were slightly acidic and of low salinity, dominated by Ca^2+^, Mg^2+^ and Na^+^; Lake samples were SO_4_^2−^–S rich and Si poor, while the Polygon and Stream samples were Si rich and SO_4_^2−^–S poor (Table 1). Although the synchrony-through-time analysis indicates that time of sampling can be important in defining a given sample’s biogeochemistry, there were no strong seasonal trends in the aquatic biogeochemistry in either 2013 or 2014, or between years. The study waters did not show clear evidence of enhanced permafrost thaw of the mineral sub-soils, or of thermokarst activity (i.e. thermal detachment slides) delivering mineral-derived solutes to the aquatic system, during the two study seasons (2013 and 2014).

Dissolved organic C is the dominant C species across all water types, followed by dissolved inorganic C and then dissolved CO_2_–C; dissolved CH_4_–C, whilst highly variable, generally only occurred at very low concentrations in the study waters. Total C concentrations were present in the following order of decreasing magnitude across the water types: Polygon > Stream > Lake, suggesting that different water types carry and process significantly different C loads. As such, it is important to consider all forms of C that may be stored or released from the region, given that C may be rapidly transformed from one form to another, and that C measurements in far downstream locations are unlikely to be representative of C processing in the upper catchment.

The analyses presented here support our original hypothesis, demonstrating the geochemical uniqueness of the water types identified as being controlled by both hydrological and biogeochemical processing. However, further studies on small catchments similar to the one presented here are vital to analyse and detect change in these biogeochemical processes in vulnerable and disturbed systems. While there are several existing small catchment studies in the Arctic, they are generally associated with regions that are currently undergoing considerable change (e.g. Abbott et al. 2015; Vonk et al. 2013). The dataset presented here, however, shows no significant biogeochemical evidence of a shift to deeper water sources linked to enhanced summer active layer deepening, with most biogeochemical variables remaining relatively stable throughout the two study seasons despite substantial (seasonal) deepening of the active layer during the snow free season. This suggests that in the absence of enhanced permafrost thaw, the aquatic biogeochemistry of the study area (and similar regions elsewhere in the Arctic) could be expected to remain relatively stable.

Although there has been marked warming and shrubification in the region in the last three decades, this study appears, nonetheless, to provide a “baseline” surface water analysis for the western Canadian Arctic from which to consider potential future changes to the region under predicted climate change. These might include (1) increased CO_2_ concentrations relative to DOC as a result of aquatic mineralisation of organic matter (2) increased DIC relative to DOC as a result of substantial deepening of the active layer such that flow paths develop within the mineral layers, and (3) a change in the total C balance of the catchments. With the caveat that we only report two years of data (2013 and 2014), this study appears to provide a good example of an Arctic system in a state of relative biogeochemical equilibrium compared to many other research sites.
